# Environmental Risk Assessment of Vehicle Exhaust Particles on Aquatic Organisms of Different Trophic Levels

**DOI:** 10.3390/toxics9100261

**Published:** 2021-10-13

**Authors:** Konstantin Pikula, Mariya Tretyakova, Alexander Zakharenko, Seyed Ali Johari, Sergey Ugay, Valery Chernyshev, Vladimir Chaika, Tatiana Kalenik, Kirill Golokhvast

**Affiliations:** 1 Polytechnical Institute, Far Eastern Federal University, 10 Ajax Bay, Russky Island, 690922 Vladivostok, Russia; tretyakova.m.o@yandex.ru (M.T.); ugay.sm@dvfu.ru (S.U.); chernyshev.vv@dvfu.ru (V.C.); golokhvast@sfsca.ru (K.G.); 2 Federal Research Center the Yakut Scientific Center of the Siberian Branch of the Russian Academy of Sciences, 2, Petrovskogo Str., 677000 Yakutsk, Russia; 3 Siberian Federal Scientific Center of Agrobiotechnologies of the Russian Academy of Sciences, SFSCA RAS, P.O. Box 267, 630501 Krasnoobsk, Russia; rarf@yandex.ru (A.Z.); chaika.vladimr@yandex.ru (V.C.); 4 Laboratory of Supercritical Fluid Research and Application in Agrobiotechnology, The National Research Tomsk State University, 36, Lenin Avenue, 634050 Tomsk, Russia; 5 Department of Fisheries, Faculty of Natural Resources, University of Kurdistan, Pasdaran St, Sanandaj 66177-15175, Iran; sajohari@gmail.com; 6 Institute of Life Science and Biomedicine, Far Eastern Federal University, 10 Ajax Bay, Russky Island, 690922 Vladivostok, Russia; kalenik.tk@dvfu.ru

**Keywords:** algae, aquatic toxicity, bioassay, ecotoxicology, particulate matter, sea urchin, ultrafine particles

## Abstract

Vehicle emission particles (VEPs) represent a significant part of air pollution in urban areas. However, the toxicity of this category of particles in different aquatic organisms is still unexplored. This work aimed to extend the understanding of the toxicity of the vehicle exhaust particles in two species of marine diatomic microalgae, the planktonic crustacean *Artemia salina*, and the sea urchin *Strongylocentrotus intermedius*. These aquatic species were applied for the first time in the risk assessment of VEPs. Our results demonstrated that the samples obtained from diesel-powered vehicles completely prevented egg fertilization of the sea urchin *S. intermedius* and caused pronounced membrane depolarization in the cells of both tested microalgae species at concentrations between 10 and 100 mg/L. The sample with the highest proportion of submicron particles and the highest content of polycyclic aromatic hydrocarbons (PAHs) had the highest growth rate inhibition in both microalgae species and caused high toxicity to the crustacean. The toxicity level of the other samples varied among the species. We can conclude that metal content and the difference in the concentrations of PAHs by itself did not directly reflect the toxic level of VEPs, but the combination of both a high number of submicron particles and high PAH concentrations had the highest toxic effect on all the tested species.

## 1. Introduction

Over the past few decades, vehicle-emitted particles (VEPs) have received much attention due to their possible negative influence on human health and the environment [1,2]. It was shown that vehicle emissions are the main source of ambient particles in urban areas [3,4,5]. Human exposure to urban particles was linked to an increase in cancer, cardiovascular, and respiratory diseases [6,7,8,9].

Environmental processes, gravitational settling, and surface wash can bring VEPs into aquatic ecosystems [10]. Many cities with high road traffic are located near seashores, which increases the risks of marine pollutions by VEPs and requires a stringent algorithm of air quality monitoring and regulation in terms of environmental and human safety [11]. Vehicle emissions could be a substantial source of metal and polycyclic aromatic hydrocarbon (PAH) contamination of marine ecosystems [12,13]. PAHs are indicated as one of the major contributors to the toxic effect of motor vehicle exhausts because of their mutagenic, carcinogenic, and teratogenic properties [14]. Moreover, the number and the characteristics of VEPs depend on many factors, such as the engine type, fuel type, mileage, and working conditions [15,16]. Many research groups have aimed to find technological solutions which reduce the emission of particulate matter (PM) by motor vehicles [17,18]. At the same time, modern types of engine and the use of alternative types of fuel can lead to incomplete fuel combustion and subsequently to an increase in PM emissions [1,19,20]. All these factors made the risk assessment and regulation of VEPs difficult.

The application of species from different trophic levels in bioassays plays a crucial role in the complex understanding of the potential environmental threat of common pollutants [21,22]. Moreover, it will have a beneficial effect on the development and revision of the standards and limitations regarding the studied contaminants. Previously, the toxicity of PM emitted by different types of light-duty vehicles was assessed in several works by a standard ecotoxicity test with *Vibrio fischeri* bacteria [23,24]. It was shown that gasoline VEPs were 12 times more toxic on a per mass basis than the PM emitted by the vehicle with a conventional diesel engine, but diesel-powered vehicles had a much higher level of particulate emissions [25]. The comparison between the emissions of diesel engines of Euro0–Euro4 standards detected no ecotoxic and genotoxic effects of particulates from Euro4 vehicles in a bioassay with *Vibrio fischeri*, contrary to vehicles with engines of the previous standards. The other study assessed the aquatic toxicity of extracts obtained from diesel PM in bacteria, microalgae, daphnids, and fish [26]. It was demonstrated that the toxicity of PM emitted by diesel cars depends on the contaminants adsorbed at their surface, and the sensitivity of the species used was as follows: daphnids > algae > bacteria > fish. However, the number of research works related to the influence of VEPs on the aquatic environment is limited [14,27] and the toxicity of VEPs in aquatic organisms are not fully understood. This field requires further investigation aimed to determine the specific parameters of VEPs, which will allow the application of regulatory measures to minimize the influence of vehicle emissions on the aquatic environment.

The multispecies approach has primary importance in the risk assessment of toxic substances in the aquatic environment [28]. The importance of diatom microalgae as an indicator of pollution is widely known [29]. Moreover, microalgae are known as the main producers of organic matter in the aquatic environment [30]. The crustacean is another common test model used in ecotoxicology [31,32]. The brine shrimp *A. salina* is a good indicator of water quality due to its high capacity for bioaccumulation of xenobiotics [33]. Moreover, it can demonstrate the potential of pollution to transfer through aquatic food chains. The embryos of sea urchins are a sensitive and reliable indicator of marine pollutions [34,35]. Sea urchin sperm and embryo–larval bioassays are efficiently applied in ecotoxicity bioassays due to their sensitivity to very low concentrations of pollutants in seawater [36].

This study aimed to compare the toxicity of exhaust particles emitted by motorcycles, light-duty vehicles, and a specialized vehicle with gasoline and diesel engines in aquatic organisms of different trophic levels, namely the diatom microalgae *Attheya ussuriensis* and *Chaetoceros muelleri*, the brine shrimp *Artemia salina*, and embryos of the sea urchin *Strongylocentrotus intermedius*. To the best of the authors’ knowledge, these species have never been used in the risk assessment of VEPs. Therefore, this work will support the understanding of the impact of VEPs and their characteristics, such as particle size and number and the concentration of toxic metals and PAHs, on different aquatic species.

## 2. Materials and Methods

### 2.1. Collection and Characterization of Vehicle Exhaust Particles

The types of vehicle used in this study were selected according to the Russian classification ON025270-66. The characteristics of the vehicles used are given in Table 1. To avoid specifying the car manufacturers and models, we applied code names to the vehicles used. All the chosen vehicles had a mileage between 50,000 and 150,000 km.

The VEPs were obtained according to the previously described method of exhaust gas suspension collection [38]. This method was approved on a wide range of different types of vehicles [39,40].

The stock suspensions of each VEP sample at the concentration of 1000 mg/L were prepared and analyzed with a laser particle sizer (Analysette 22 NanoTec plus, Fritsch, Germany) as described in our previous work [27]. For each sample, the percentage of particles in a certain size range was calculated based on five measurements.

The optical density of prepared stock suspensions of the VEP samples was measured by an Epoch UV-Vis microplate spectrophotometer (BioTek Instruments, Inc., Winooski, VT, USA) at a wavelength of 860 nm.

Polycyclic aromatic hydrocarbons (PAHs) in the methanol and water-soluble fractions of the VEPs were analyzed by a single quadrupole gas chromatograph-mass spectrometer (Shimadzu QP2010) equipped with a split/splitless injection inlet and an AOC-5000 auto-sampler as previously described [41].

The metal content was measured in the stock suspensions of VEP after 7 days of suspension preparation. This analysis was performed with an ICP-MS spectrometer (Agilent 7700x, Agilent Technologies, Santa Clara, CA, USA).

The morphology of the obtained particles was studied by scanning electron microscope Carl Zeiss Ultra 55 (Carl Zeiss, Oberkochen, Germany).

### 2.2. Microalgae Bioassay

#### 2.2.1. Microalgae Cultures

Microalgal cultures were provided by The Resource Collection Marine Biobank of the National Scientific Center of Marine Biology, Far Eastern Branch of the Russian Academy of Sciences (NSCMB FEB RAS). The bioassays were conducted on two marine diatoms (Bacillariophyta), namely *A. ussuriensis* (Stonik, Orlova et Crawford, 2006) and *C. muelleri* (Lemmermann, 1896) (Figure S1).

Microalgae cultivation and toxicity tests were conducted according to the guidance of OECD No. 201 [42] with minor modifications, as described previously [43]. For microalgae bioassays, we used 24-well plates with VEPs at the concentrations of 1, 10, and 100 mg/L. The working suspensions of VEPs were sonicated with an ultrasound homogenizer (Bandelin Sonopuls GM 3100, Bandelin Electronic GmbH & Co. KG, Berlin, Germany) with a high-frequency power of 100 W for 30 min before each series of bioassays. The sonication was performed to prevent initial particle agglomeration, according to the protocols of particle suspension testing [44] and previous work [27,45]. The wells with only the f/2 medium [46] were taken as a control group. All the bioassays were performed in quadruplicate. The volume of microalgae aliquots in each well was 2 mL.

#### 2.2.2. Flow Cytometry Analysis

Registration of the state of the microalgae cells during the experiment was carried out with a flow cytometer (CytoFLEX, Beckman Coulter, Indianapolis, IN, USA) with the software package CytExpert v.2.0. Biochemical changes in the microalgae cells after exposure to the VEPs were evaluated using specific fluorescent dyes (Table 2). Each sample was measured at a flow rate of 100 μL/min for 30 s, according to previous work [27,45]. The blue laser (488 nm) of the CytoFLEX flow cytometer was chosen as the source of excitation light. The excitation source and emission channels were selected based on the data provided by the manufacturer of the dyes (Molecular Probes, Eugene, OR, USA). All the data of the flow cytometric measurements were registered and collected as the mean fluorescence intensity. The endpoints used in this work, including the registration time, fluorescent dyes used, and emission channels are listed in Table 2. The procedure of optimizing the concentration of the fluorescent dyes used for each microalgae species was described in previous work [47].

### 2.3. Brine Shrimp Bioassay

The zooplanktonic crustacean *A. salina*, also known as brine shrimp, was used to assess the toxic impact of the obtained VEPs. The bioassays were performed according to the standards of nanomaterial toxicity testing on brine shrimp nauplii ISO/TS 20787:2017 [51]. Brine shrimp eggs were purchased locally and hatched in filtered (pore diameter: 0.22 μm) sterile seawater with 33 ± 1‰ salinity and pH 8.0 ± 0.2. The cultivation was carried out at a temperature of 20 ± 2 °C with an illumination intensity of 300 μmol photons/m^2^ s, with a light cycle of 16:8 h.

The exposure of brine shrimp nauplii to the VEPs was carried out in 96-well plates with the VEPs at concentrations of 50, 100, and 250 mg/L. The volume of tested liquid in each replication was 200 µL. The wells with only seawater were taken as a control group. Newborn nauplii (24–36 h) were transferred to a 96-well plate (10 animals per well). All the assays for each concentration of VEPs and the control group were performed in quadruplicate. The counting of dead and alive animals was performed after 24, 48, and 96 h of exposure. Morphological changes of *A. salina* nauplii exposed to the VEPs were captured by an optical microscope (Axio Observer A1, Carl Zeiss, Jena, Germany).

### 2.4. Sea Urchin Bioassay

The protocol of the sea urchin bioassay has been described previously [34]. Adult individuals of the sea urchin *S. intermedius* were collected from Novi Dzhigit Bay (Peter the Great Bay, Sea of Japan, Russia). The genital products of male and female urchins received by the modified method of Buznikov [52] were washed and diluted with sterilized (heating in a microwave for 10 min) filtered seawater (filter pore: 0.22 μm). The quality of the obtained material was controlled by trial fertilization of the eggs. More than 99.8% of the cells (Figure S2a) formed a fertilization membrane (Figure S2b).

The toxicity of the VEPs was evaluated by two types of tests with the sea urchin model, namely (1) egg fertilization inhibition and (2) the early-stage embryo development test. The incubation was carried out in 24-well plates at a temperature of 18–20 °C.All the experiments were performed in quadruplicate. For each replication, at least 100 cells were observed by an inverted optical microscope (Axio Observer Z1, Carl Zeiss, Oberkochen, Germany) in cell aliquots of 1000 μL. The VEPs, at a volume of 100 μL VEPs, were added to 900 μL aliquots of sea urchin cells to obtain final concentrations of 10, 25, 50, and 100 mg/L. Before each series of bioassays, the working suspensions of VEPs were sonicated with an ultrasound homogenizer (Bandelin Sonopuls GM 3100, Bandelin Electronic GmbH & Co. KG, Berlin, Germany). In total, 100 μL of sterile seawater was added to the control groups.

Egg fertilization inhibition was measured by half an hour of exposure of an 800 μL aliquot of unfertilized eggs (2 × 10^3^ cells/mL) to the VEPs, with the further addition of 100 μL of the spermatozoa (1 × 10^8^ cells/mL). The percentage of fertilized eggs was calculated relative to the total number of eggs counted.

Embryo development was assessed by the addition of VEPs to the eggs of *S. intermedius* 5 min after fertilization. The fertilization was performed as described above. The embryos that reached correct development in 24 h were defined as normal. The features of normal development were described previously [34]. The number of normal embryos (Figure S2c), the embryos with developmental delays or impaired development (Figure S2d), and dead embryos were counted relative to the total number of zygotes.

### 2.5. Statistical Analysis

Statistical analyzes were performed using GraphPad Prism 8.0.2 (GraphPad Software, San Diego, CA, USA). Normality was checked using the Shapiro–Wilk test. One-way ANOVA tests were used for analysis. A value of *p* ≤ 0.05 was considered statistically significant.

## 3. Results

### 3.1. Characteristics of the Obtained Vehicle Emitted Particles

**Figure 1:**
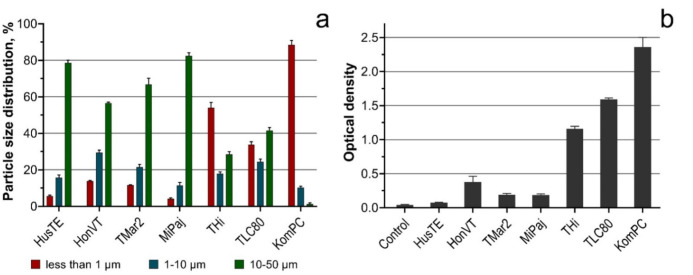
Characteristics of the VEP samples: (**a**) particle size distribution; (**b**) optical density.

The results of the laser granulometry analyses are presented in Figure 1a. The optical density of the VEP suspensions is given in Figure 1b. According to the laser granulometry data (Figure 1a), the VEPs samples obtained from diesel-powered vehicles (i.e., THi, TLC80, and KomPC) had a higher number of particles in the size range of less than 1 µm compared with all the other samples. The results of the optical density measurement (Figure 1b) revealed the highest light absorption in the same samples (THi, TLC80, and KomPC). The correlation between the optical density of the suspensions and the particle size distribution was evaluated by computing the Pearson correlation coefficients. The analysis showed that the optical density significantly depended (*p* = 0.002) on the number of particles in the size ranges of less than 1 µm and 10–50 µm. Therefore, the higher share of particles less than 1 µm, the higher the optical density of the suspension, and vice versa for the share of particles in the size range of 10–50 µm, i.e., the higher share of the particles in the size range of 10–50 µm, the lower the optical density of the suspension. The number of particles in the size range of 1–10 µm had no significant effect (*p* > 0.05) on the optical density of the suspensions.

The PAH content in the stock suspensions of VEP is displayed in Table 3. The highest PAH content was registered in the KomPC sample (>100 ng/mg). Moderate concentrations (>10 ng/mg) were registered for THi and HusTE.

The metal content of the stock suspensions of VEP is displayed in Table 4. Among the registered chemical species, all the analyzed samples had a high concentration of strontium, because it is used as a catalyst in vehicle exhaust systems. The HonVT sample can be highlighted for its higher concentrations of aluminum, manganese, and nickel compared with the other VEP samples. The TMar2 sample had the highest concentration of molybdenum; THi had a prevailing value of zinc. The TLC80 sample had slightly higher concentrations of chromium, lead, and cadmium. At the same time, KomPC had a high concentration of iron.

The morphology of the particles emitted by gasoline-driven vehicles obtained by scanning electron microscopy is presented in Figure S3. The morphology of the particles emitted by diesel-driven vehicles is presented in Figure S4.

### 3.2. Results of the Microalgae Bioassay

**Figure 2:**
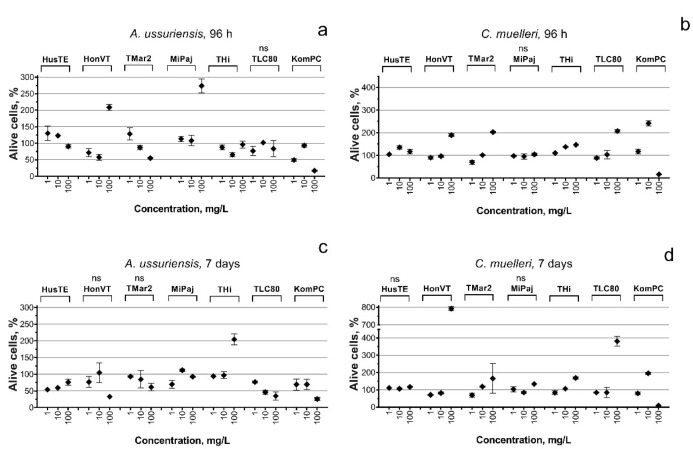
The influence of VEP samples on the microalgae growth rate: (**a**) *A. ussuriensis* after 96 h of exposure; (**b**) *C. muelleri* after 96 h of exposure; (**c**) *A. ussuriensis* after 7 days of exposure; (**d**) *C. muelleri* after 7 days of exposure; ns, the tested sample had no significant effect on the growth rate of microalgae (*p* > 0.05). The series without the mark “ns” significantly influenced the growth rate of the microalgae (*p* < 0.05).

For the samples of VEPs which revealed a toxic effect towards the microalgae species used, we computed the concentrations that caused 50% growth rate inhibition in microalgae cells (EC50) compared with the control (Table S1). Both microalgae species demonstrated the highest growth rate inhibition after exposure to the KomPC sample. The 24 h and 96 h EC50 values for both microalgae species exposed to this sample were in a range between 43.5 and 61.8 mg/L. Moreover, this sample demonstrated chronic toxicity (the 7 day EC50 concentration was lower than the 24 and 96 h values). Only the KomPC sample revealed both acute and chronic toxicity for both microalgae species used. The TLC80 sample revealed chronic toxicity for *A. ussuriensis* only. It should be noted that the KomPC and TLC80 samples were obtained from vehicles powered by diesel fuel (Table 1). The other tested samples either had no significant influence on the growth rate of microalgae or they stimulated the growth rate (Figure 2). The most pronounced growth rate stimulation (up to eight times) was observed for *C. muelleri* exposed to the HonVT sample for 7 days (Figure 2d). At the same time, the HonVT sample had no significant effect on the growth rate of *A. ussuriensis* after 7 days of exposure (Figure 2c). The most pronounced growth rate stimulation of *A. ussuriensis* was observed after 96 h of exposure to the MiPaj sample (Figure 2a) and after 7 days of exposure to the THi sample (Figure 2c).

**Figure 3:**
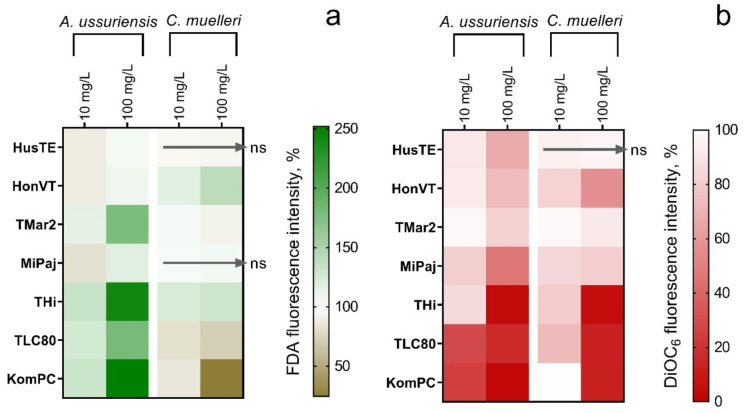
The changes in esterase activity and membrane polarization of the diatomic microalgae *A. ussuriensis* and *C. muelleri* after 24 h of exposure to the VEPs, (**a**) Esterase activity changes; (**b**) membrane polarization changes. ns, the tested sample had no significant effect on the esterase activity or membrane potential of microalgae cells (*p* < 0.05). The concentration of 1 mg/L of all the tested samples had no significant effect on the esterase activity and membrane potential of microalgae (not represented on the graph).

The changes in the esterase activity and membrane polarization of microalgal cells after 24 h of exposure to the VEPs are represented in a heatmap (Figure 3). The pronounced inhibition of esterase activity was detected for the cells of *C. muelleri* microalgae only under exposure to the TLC80 and KomPC samples (Figure 3a). The cells of *A. ussuriensis* responded with stimulation of esterase activity under the influence of the samples TMar2, THi, TLC80, and KomPC (Figure 3a). The HusTE and MiPaj samples had no significant influence on the esterase activity of *C. muelleri*, which correlates with the lowest content of metals found in these samples (Table 4).

Most of the VEP samples induced membrane depolarization in microalgae cells (Figure 3b). The THi, TLC80, and KomPC samples demonstrated the highest effect among the samples (Figure 3b). At the same time, the HusTe sample had no significant influence on the membrane polarization of *C. muelleri*. In general, all the VEP samples caused dose-dependent membrane depolarization in microalgae cells.

**Figure 4:**
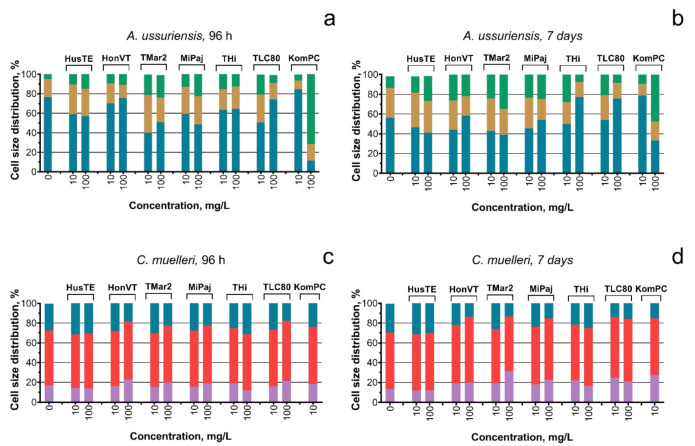
The size distribution of microalgae cells exposed to the VEPs: (**a**) *A. ussuriensis* after 96 h of exposure; (**b**) *A. ussuriensis* after 7 days of exposure; (**c**) *C. muelleri* after 96 h of exposure; (**d**) *C. muelleri* after 7 days of the exposure. The results for the measurement with *C. muelleri* exposed to 100 mg/L of the KomPC sample are not represented in the graph because most *C. muelleri* cells were dead during the measurement because of the high toxicity of this sample. The concentration of 1 mg/L of all the tested samples had no significant effect on the size of microalgae cells (not represented on the graph).

The influence of the VEP samples tested on the changes in the size of microalgae cells is presented in Figure 4. Almost all the samples caused enlargement of *A. ussuriensis* cells compared with the control (Figure 4a,b). The highest effect on the cells of *A. ussuriensis* was for the KomPC sample at the concentration of 100 mg/L. *C. muelleri* revealed no significant size changes after exposure to the VEPs.

### 3.3. Results of the Brine Shrimp Bioassay

**Figure 5:**
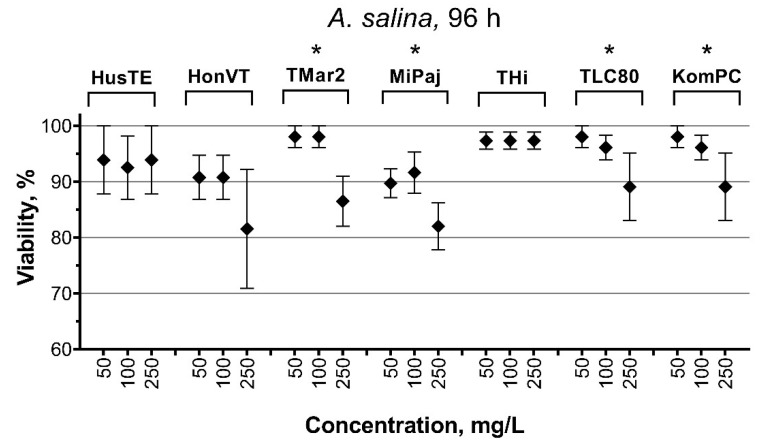
The influence of the VEPs on the viability of *A. silina* nauplii after 96 h of exposure compared with the control (100%). * *p* < 0.05.

The results of the bioassay with brine shrimp nauplii showed neither immobilization nor lethal cases, even at the highest concentration of the VEPs (250 mg/L) after 24 and 48 h of exposure. The mortality of *A. silina* nauplii after 96 h of exposure to the VEPs is presented in Figure 4. Only the MiPaj sample caused the mortality of *A. silina* at the concentrations of 50 and 100 mg/L (Figure 5). At the concentration of 250 mg/L, all the tested VEP samples except THi caused mortality in between 10 and 30% of the nauplii. The THi and HusTE samples demonstrated the lowest toxicity to the brine shrimp nauplii compared with the other VEP samples.

The microscopic observation of *A. silina* after 96 h of exposure showed pronounced uptake and absorption of the agglomerated VEPs in the guts of the nauplii (Figure S5).

### 3.4. Results of the Sea Urchin Egg Fertilization and Embryotoxicity Tests

The results of the sea urchin egg fertilization test revealed no contraceptive activity after the influence of the samples obtained from gasoline-powered vehicles (HusTE, HonVT, TMar2, and MiPaj). However, the samples obtained from diesel-powered vehicles (THi, TLC80, and KomPC) almost completely prevented egg fertilization and made the eggs sterile. No fertilization was registered even after the addition of a new portion of the spermatozoa (100 μL, 1 × 10^8^ cells/mL). Moreover, the optical microscopy demonstrated adsorption of the THi, TLC80, and KomPC samples to the surface of the embryos (Figure S6).

**Figure 6:**
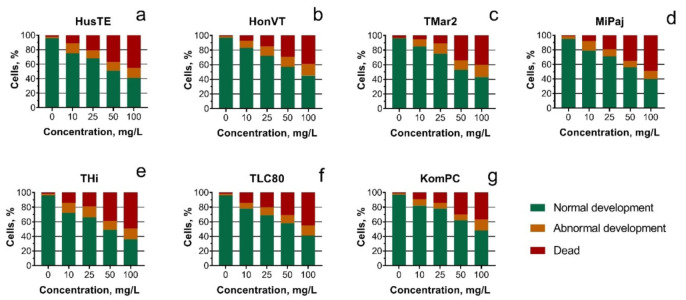
The state of *S. intermedius* embryo development after 24 h of the exposure to the tested VEP samples: (**a**) HusTE; (**b**) HonVT; (**c**) TMar2; (**d**) MiPaj; (**e**) THi; (**f**) TLC80; (**g**) KomPC.

The concentrations that caused 50% of embryo mortality (LC50) were calculated for all the VEPs samples (Table 5). The highest toxicity was registered for the THi sample. However, in general, all the tested samples had a comparable level of toxicity. The changes in embryo development after exposure to the VEPs are shown in Figure 6. All the VEP samples caused a similar level of developmental disorder in the sea urchin embryos.

## 4. Discussion

It should be summarized that the tested samples of VEPs had different levels of toxicity depending on the aquatic species used. Our previous work [27] demonstrated that the particles emitted by diesel-powered vehicles (KomPC, THi, and TLC80) had the highest toxic effect on the growth rate of the red alga *Porphyridium purpureum* and the Raphidophycean alga *Heterosigma akashiwo*. However, in contrast to previous results [27], the THi sample increased the growth rate of both diatomic microalgae species used in the current study (Figure 2c,d) and the TLC80 sample increased the growth rate of *C. muelleri* (Figure 2d) after 7 days of exposure. It is noteworthy that in the abovementioned cases, the lower concentrations of the VEPs (1 and 10 mL/L) had no influence on the growth rate of microalgae cells, and the highest concentration used (100 mg/L) caused stimulation of microalgal growth rate. This observation fits the biphasic dose–response relationship called hormesis [53], which has commonly been observed in plants [54]. Therefore, the concentrations of most of the tested VEP samples did not reach the level of adverse effects in the microalgae species used but reached stimulation levels according to a hormetic dose–response curve. Thus, only the KomPC sample reached the level of adverse effects in both microalgae species (Figure 2). This sample had the highest percentage of particles in the size range of less than 1 μm (Figure 1a) and a higher PAH concentration (Table 3). A review [55] demonstrated that fine PM smaller than 1.1 μm has significantly higher health and environmental exposure compared with bigger particles because of the higher accumulation of PAHs, which is correlated with the observations of the current study. Hence, a high number of particles of less than 1 μm and the high PAH content in the VEP of the KomPC sample are the most probable reasons for its high toxicity in the tested species. However, the increase in the microalgal growth rate caused by lower concentrations of VEPs can be a reason for algal blooms and further negative impacts on other aquatic organisms [56,57].

The high number of submicron particles (Figure 1a) in the THi, TLC80, and KomPC samples reflect the highest membrane depolarization in the cells of both microalgae species (Figure 3b), the mortality of *A. silina* nauplii (Figure 4 and Figure S5), and the inhibition of sea urchin egg fertilization (Table 5). It is known that the adsorption of particles to aquatic organisms increases the role of physical damage as the most probable mechanism of toxicity [58].

Interestingly, *A. ussuriensis* responded with an increase in esterase activity and *C. muelleri* responded with inhibition of esterase activity in response to the influence of the KomPC sample (Figure 3). Both cases indicate the disruption of metabolic activity of the cells and might be the reason for further responses to the toxic influence. The disruption of intracellular enzyme activity (esterase) can be assumed to be an important indicator of sublethal toxicity in microalgae [49,59].

Unlike the microalgae and sea urchin bioassay, the relatively high mortality of *A. salina* nauplii was observed under the influence of the samples from gasoline-powered vehicles, namely TMar2 and MiPaj. The nauplii of *A. silina* demonstrated tolerance to the VEP of the THi sample (Figure 4), despite the high membrane depolarization in microalgae cells (Figure 3b) and the high toxicity in sea urchin embryo development (Table 5) and egg fertilization tests. These results indicate the different sensitivity of aquatic species, which strongly depends on the permeability and bioavailability of the toxins or their combination to the cells or organisms. Previously, the differences in the bioavailability of benzo(a)pyrene to brine shrimp were shown in a combined exposure with different types of carbon-based nanomaterials [60].

The higher content of toxic metals such as zinc and arsenic (Table 4) might be the reason for the high toxicity of the THi sample to the embryos of the sea urchin *S. intermedius* (Table 5). Despite zinc being an essential metal, its toxicity has been reported in many aquatic organisms, including crustaceans [61,62]. Therefore, we can assume that in the case of sea urchin embryotoxicity, the factor of the chemical composition of PM was more important than the size of the particles. Moreover, the KomPC sample, which was the most toxic for microalgae, had the lowest toxic influence on the embryos of the sea urchin *S. intermedius* (Table 5). These results might be associated with the different permeability and bioavailability of VEPs for different types of cells.

According to the obtained data, we can infer that the environmental risk assessment and regulation of VEPs should be considered as heterogeneous multicomponent mixtures of toxins, which require the development and application of standard protocols. Recent toxicological works have focused on studying the toxicity of mixtures and the application of a multispecies approach in risk assessment [63,64]. The same approach should be applied to the regulation of vehicle emissions. The different sensitivity and different responses of the model species used to the tested VEP samples represent a significant interest for further research. Further study is required for an understanding of the mechanisms of toxic action of VEPs to aquatic organisms and the environment. This knowledge will allow the development of standard protocols of bioassays and predictive computational algorithms, and finally, it will lead to regulatory measures aimed at maintaining a safe environment.

## 5. Conclusions

The present research aimed to examine the aquatic toxicity of the PM emitted by seven vehicles on aquatic species, namely the diatomic microalgae *A. ussuriensis* and *C. muelleri*, the brine shrimp *A. salina*, and the sea urchin *S. intermedius*. Our study demonstrated the higher toxicity of VEPs obtained from diesel-powered vehicles, compared with VEPs obtained from gasoline-powered vehicles, in a microalgae bioassay and a sea urchin egg fertilization bioassay. However, this correlation cannot be applied to the brine shrimp mortality and sea urchin embryo development tests. Moreover, only the samples obtained from diesel-powered vehicles completely prevented egg fertilization of the sea urchin *S. intermedius* and caused pronounced membrane depolarization in the cells of both microalgae species used.

Although this study does not provide the mechanisms of toxic action of the tested VEPs samples in the species used, some general correlations can be highlighted. We can indicate that the size and number of the particles play one of the most important roles in the toxic action of VEPs towards microalgae and sea urchin eggs, i.e., a higher number of submicron particles can indicate the higher toxicity of the emissions. At the same time, the content of toxic metals and PAHs by itself does not directly show the highly toxic action of tested VEPs and depends on the sensitivity of different aquatic organisms to the toxic action of specific components. However, the combination of a high number of submicron particles and high PAH concentrations had the most pronounced toxic effect on all the tested species.

The aquatic species were applied for the first time in the risk assessment of VEPs, which serveed to obtain a better understanding of their toxic action in the aquatic environment. Further studies with the application of an extended set of toxicity endpoints and a more comprehensive protocol of bioassays are required for understanding the mechanisms of toxic action of VEPs and their individual components to aquatic organisms and the environment.

## Figures and Tables

**Table 1:** List of the vehicles used in the experiment.

Vehicle Type	Coded Vehicle Model	Displacement (cc)	Fuel (Russian Standard) ^1^
Motorcycle	HusTE	300	AI–92
	HonVT	1300	AI–95
Light-duty vehicle	TMar2	2500	AI–92
	MiPaj	3000	AI–95
	THi	3000	Diesel
	TLC80	2500	Diesel
Specialized vehicle	KomPC	8300	Diesel

^1^ AI-92 and AI-95 are the codes of gasoline types according to the Russian standard GOST 2084-77, where 92 and 96 are the values of octane numbers [37].

**Table 2:** The criteria and conditions of the microalgae bioassays.

Endpoint	Exposure	Fluorescent Dye or Registered Parameter	CytoFLEX Emission Channel Name/ WAVELENGTH, nm	Reference
Growth rate inhibition	24 h, 96 h, 7 days	PI	ECD, 610	[48]
Esterase activity	24 h	FDA	FITC, 525	[49]
Membrane potential	24 h	DIOC_6_	FITC, 525	[50]
Size	96 h, 7 days	Forward scatter intensity (size calibration kit F13838 by Molecular Probes, USA)	FSC	[45]

PI, propidium iodide; FDA, fluorescein diacetate; DIOC6, 3,3′-dihexyloxacarbocyanine iodide

**Table 3:** PAH content in the stock suspensions of VEPs, µg/L.

Sample	ACEN	FLU	Sum of ANTH and PHEN	PYR	SUM of BaANTH and CHRY	SUM of BkFLU, BaFLU, and BaPYR	Sum of BghiPER and BahANTH	IND123PYR	Total PAHs
HusTE	0.00	2.56	11.64	1.56	0.05	0.02	0.00	0.00	15.83
HonVT	0.55	2.33	4.58	0.45	0.03	0.04	0.00	0.02	7.99
TMar2	0.00	1.13	4.19	0.49	0.03	0.01	0.00	0.00	5.84
MiPaj	0.20	1.85	5.70	0.37	0.01	0.03	0.14	0.28	8.59
THi	1.87	9.18	23.50	1.75	0.06	0.07	0.19	0.42	37.04
TLC80	0.06	0.23	2.06	0.43	0.04	0.01	0.00	0.04	2.86
KomPC	2.76	2.82	60.41	43.08	0.90	0.21	0.25	0.64	111.07

ACEN, acenaphtylene; FLU, fluorene; ANTH, anthracene; PHEN, phenanthrene; PYR, pyrene; BaANTH, benzo(a)anthracene; CHRY, chrysene; BkFLU, benzo(k)fluoranthene; BaFLU, benzo(a)fluoranthene; BaPYR, benzo(a)pyrene; BghiPER, benzo(g,h,i)perylene; BahANTH, dibenz(a,h)anthracene; IND123PYR, indeno(1,2,3-cd)pyrene.

**Table 4:** The results of ICP-MS analyses of VEP suspensions in seawater.

Chemical Species	Concentration in Suspension, µg/L						
	HusTE	HonVT	TMar2	MiPaj	THi	TLC80	KomPC
^27^Al	79.20	**402.90**	283.10	127.00	106.10	229.70	95.40
^45^Sc	≤0.19	≤0.14	≤0.15	≤0.20	≤0.10	≤0.18	≤0.24
^51^V	1.18	0.54	1.47	1.00	0.85	0.31	0.29
^52^Cr	≤1.70	≤1.40	≤1.80	**5.00**	≤1.90	**5.20**	≤2.20
^55^Mn	163.27	**722.08**	122.39	289.00	38.74	22.97	35.18
^56^Fe	20.32	30.00	17.95	≤13.00	29.12	40.94	**63.59**
^59^Co	1.04	1.59	3.32	1.00	3.92	0.77	3.77
^60^Ni	23.45	**280.50**	15.40	10.00	32.15	15.74	13.33
^63^Cu	67.64	66.91	71.09	73.00	68.94	78.30	74.06
^66^Zn	25.68	141.50	554.00	513.00	**852.10**	36.50	307.50
^75^As	1.41	0.90	1.81	2.00	**3.38**	1.74	0.91
^88^Sr	7234	8076	**8941**	8236	8075	**8741**	8136
^89^Y	0.06	0.03	0.04	n/a	0.03	0.06	0.06
^90^Zr	0.41	0.18	0.18	n/a	0.14	0.22	0.21
^93^Nb	≤0.02	0.03	0.02	n/a	≤0.01	0.02	≤0.02
^98^Mo	18.74	166.30	**608.70**	242.00	161.50	16.66	19.11
^107^Ag	0.42	0.11	0.06	n/a	0.17	0.06	0.04
^114^Cd	0.14	0.15	0.45	n/a	**1.64**	**2.33**	0.30
^118^Sn	0.25	0.17	0.24	n/a	0.19	0.40	0.25
^121^Sb	1.31	**3.41**	0.99	1.00	1.24	0.48	0.85
^184^W	0.16	2.37	**13.99**	n/a	0.97	0.20	0.29
^205^Tl	≤0.03	0.03	0.04	n/a	0.05	≤0.03	≤0.03
^208^Pb	0.63	0.52	0.83	1.00	1.54	**2.46**	0.49
^209^Bi	≤0.03	≤0.02	≤0.03	≤0.02	≤0.02	≤0.03	≤0.03
^232^Th	0.03	≤0.02	0.04	≤0.022	≤0.02	≤0.03	0.03
^238^U	1.55	0.76	**2.62**	2.00	0.81	0.14	0.11

The values highlighted in bold were at least one standard deviation higher than the mean value of this element registered in all the tested samples; n/a, the value was lower than the detection limit.

**Table 5:** Mean calculated LC50 values of embryo mortality of the sea urchin *S. intermedius* after 24 h of exposure to the VEPs.

Sample	HusTE	HonVT	TMar2	MiPaj	THi	TLC80	KomPC
LC50, mg/L	52.8 (35.4–79.9)	68.2 (45.2–105.8)	65.9 (50.9–86.3)	60.2 (42.7–85.9)	45.3 (28.2–73.5)	60.6 (38.6–97.5)	82.6 (57.5–122.1)

95% confidence limits are presented in th parentheses; *p* < 0.001.

## Data Availability

Not applicable.

## Supplementary Materials

The following are available online at https://www.mdpi.com/article/10.3390/toxics9100261/s1. Figure S1: Microalgae cultures used in the experiment. Figure S2: The eggs of the sea urchin *S. intermedius.* Figure S3: Scanning electron microscopy pictures of the particles emitted by gasoline vehicles. Figure S4: Scanning electron microscopy pictures of the particles emitted by diesel vehicles. Figure S5: The nauplii of *A. salina* after 96 h of the exposure to the VEPs. Figure S6: The embryos after exposure of the eggs of the sea urchin *S. intermedius* to the VEPs. Table S1: Mean calculated EC50 values of microalgae growth rate inhibition, mg/L.
