# Supplementary material for: Environmental Risk Assessment of Vehicle Exhaust Particles on Aquatic Organisms of Different Trophic Levels
Source: Toxics. 2021 Oct 13;9(10):261. doi: 10.3390/toxics9100261 (PMC8539507; doi:10.3390/toxics9100261)
Supplement: Supplementary file 1 [file toxics-09-00261-s001.zip › toxics-1404014-supplementary.pdf]

toxics

MDPI

Supplementary Materials: Environmental Risk Assessment of Vehicle Exhaust Particles on Aquatic Organisms of Different Trophic Levels

Konstantin Pikula, Mariya Tretyakova, Alexander Zakharenko, Seyed Ali Johari, Sergey Ugay, Valery Chernyshev, Vladimir Chaika, Tatiana Kalenik and Kirill Golokhvast

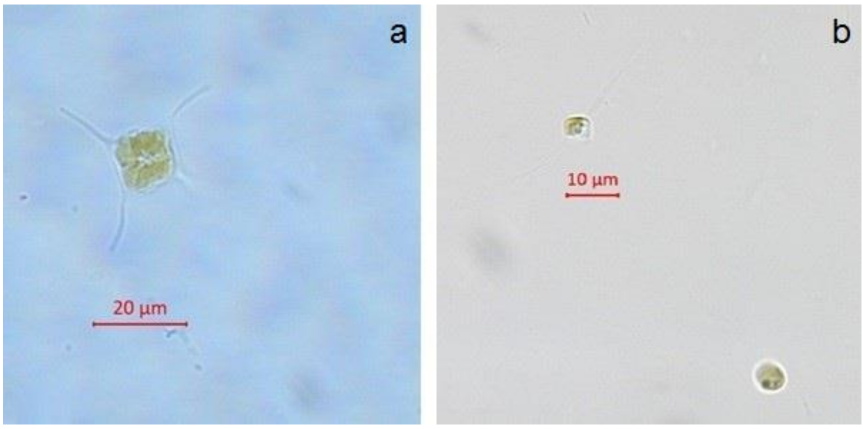

Figure S1. Microalgae cultures used in the experiment: (**a**) *A. ussuriensis*; (**b**) *C. muelleri*.

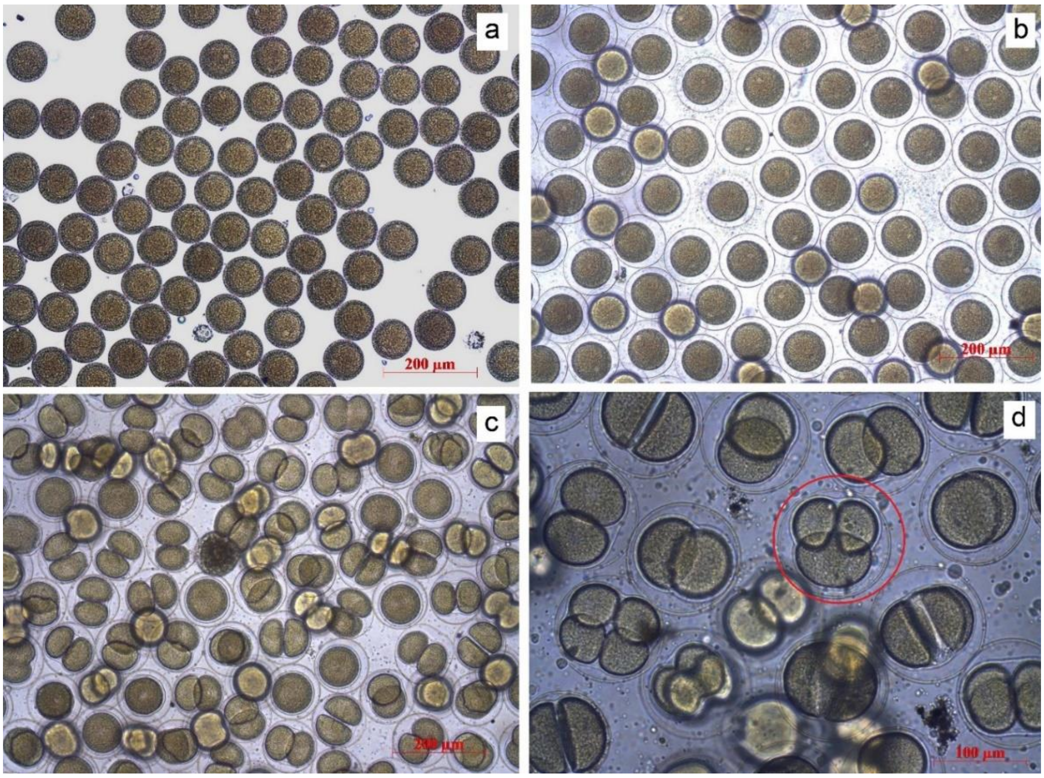

Figure S2. The eggs of sea urchin *S. intermedius*: (**a**) Sterile eggs; (**b**) Fertilized eggs; (**c**) Normal development stage of the embryos; (**d**) An example of abnormality embryo development.

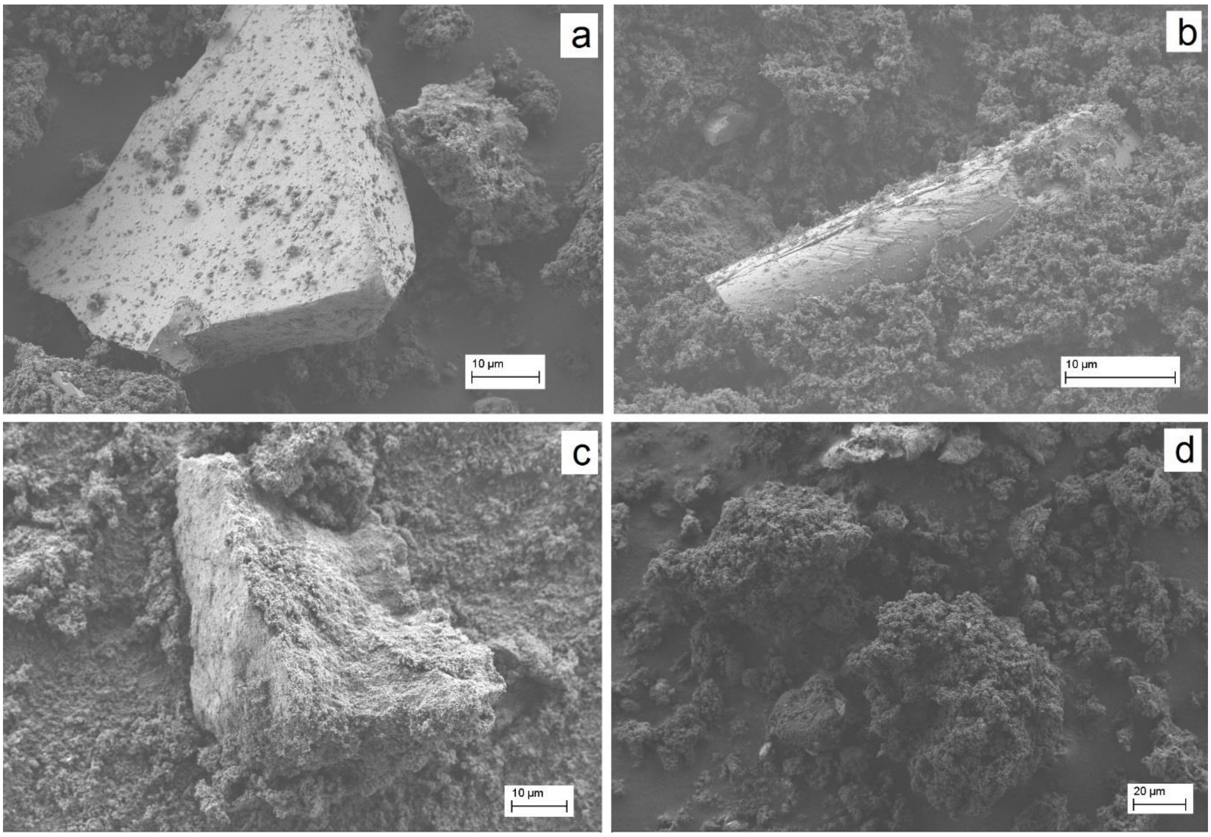

Figure S3. Scanning electron microscopy pictures of the particles emitted by gasoline driven vehicles: (**a**) HusTE; (**b**) HonVT; (**c**) TMar2; (**d**) MiPaj.

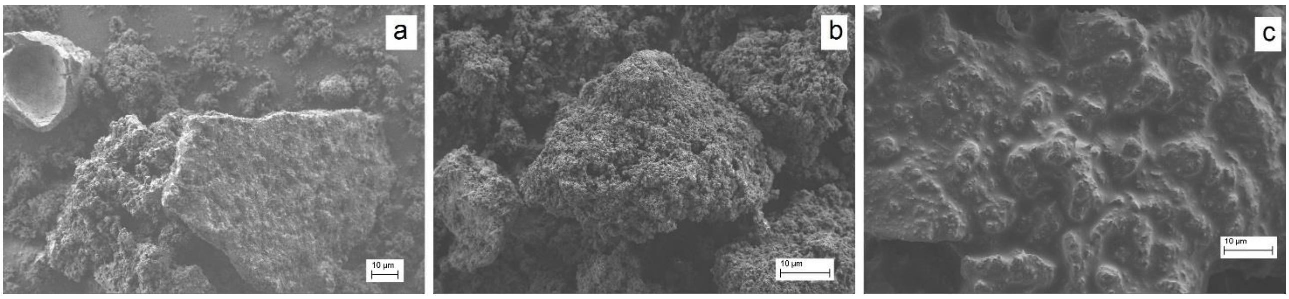

Figure S4. Scanning electron microscopy pictures of the particles emitted by diesel driven vehicles: (**a**) THi; (**b**) TLC80; (**c**) KomPC.

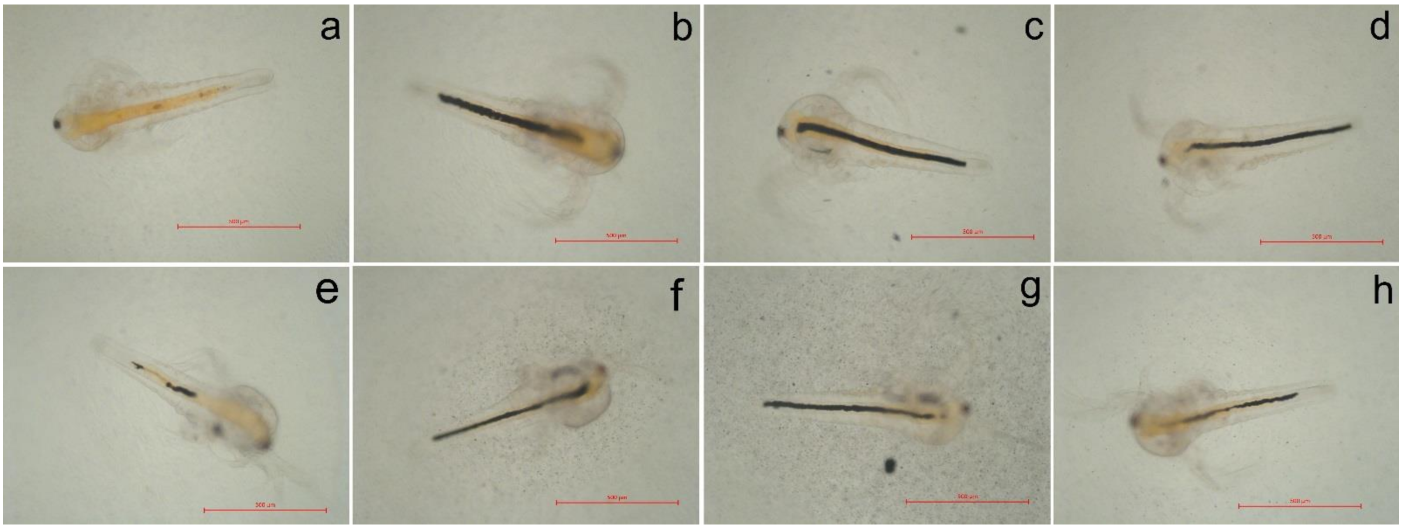

Figure S5. The nauplii of *A. salina* after 96 h of the exposure to the VEPs: (**a**) control; (**b**) HusTE; (**c**) HonVT; (**d**) TMar2; (**e**) MiPaj; (**f**) THi; (**g**) TLC80; (**h**) KomPC.

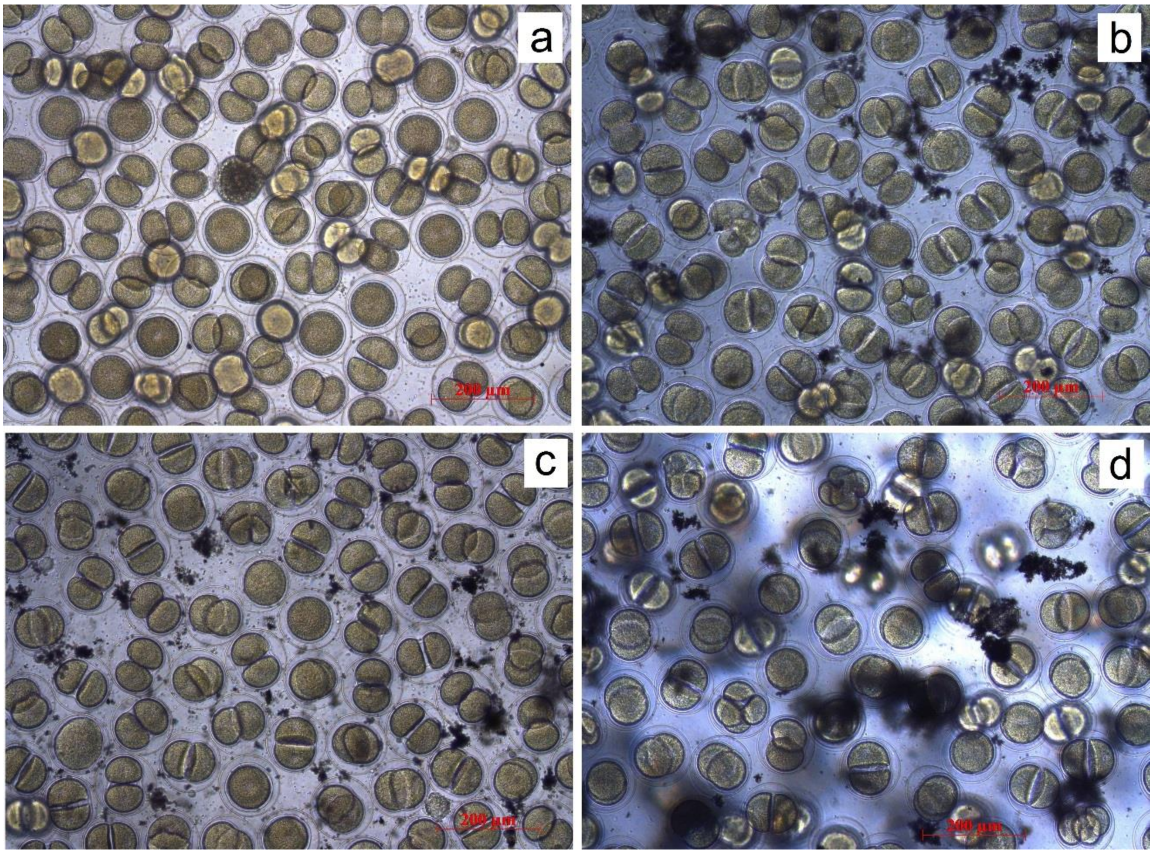

Figure S6. The embryos after the exposure of the eggs of the sea urchin *S. intermedius* to the VEPs: (**a**) control; (**b**) THi; (**c**) TLC80, (**d**) KomPC.

Table S1. Mean calculated EC50 values of microalgae growth-rate inhibition, mg/L.

**Sample**	**Exposure time**		
	**24 h**	**96 h**	**7 days**
	A. ussuriensis		
HusTE	n/a	n/a	n/a
HonVT	n/a	n/a	ns
TMar2	325.1 (130.7–554.0)	n/a	ns
MiPaj	n/a	n/a	n/a
THi	59.9 (25.3–75.1)	n/a	n/a
TLC80	ns	ns	21.1 (0.9–76.6)
KomPC	57.0 (24.15–80.34)	43.5 (29.6–57.0)	18.0 (1.2–91.8)
	C. muelleri		
HusTE	n/a	n/a	ns
HonVT	n/a	n/a	n/a
TMar2	130.2 (105.0–143.7)	n/a	n/a
MiPaj	n/a	ns	ns
THi	n/a	n/a	n/a
TLC80	n/a	n/a	n/a
KomPC	58.66 (37.4–102.0)	61.8 (30.9–86.8)	39.3 (15.1–54.2)

95% confidence intervals presented in the parentheses; n/a, the growth rate inhibition was registered at the low level, which did not allow to compute the EC50 values; ns, the tested sample had no significant effect on the growth rate of microalgae ($p > 0.05$).
